# Factors influencing precision medicine knowledge and attitudes

**DOI:** 10.1371/journal.pone.0234833

**Published:** 2020-11-11

**Authors:** Rohini Chakravarthy, Sarah C. Stallings, Michael Williams, Megan Hollister, Mario Davidson, Juan Canedo, Consuelo H. Wilkins

**Affiliations:** 1 Vanderbilt University School of Medicine, Nashville, TN, United States of America; 2 Meharry-Vanderbilt Alliance, Nashville, TN, United States of America; 3 Department of Biostatistics, Vanderbilt University School of Medicine, Nashville, TN, United States of America; 4 Department of Medicine, Division of Geriatrics, Vanderbilt University Medical Center, Nashville, TN, United States of America; Qatar University College of Medicine, QATAR

## Abstract

Precision medicine holds great promise for improving health and reducing health disparities that can be most fully realized by advancing diversity and inclusion in research participants. Without engaging underrepresented groups, precision medicine could not only fail to achieve its promise but also further exacerbate the health disparities already burdening the most vulnerable. Yet underrepresentation by people of non-European ancestry continues in precision medicine research and there are disparities across racial groups in the uptake of precision medicine applications and services. Studies have explored possible explanations for population differences in precision medicine participation, but full appreciation of the factors involved is still developing. To better inform the potential for addressing health disparities through PM, we assessed the relationship of precision medicine knowledge and trust in biomedical research with sociodemographic variables. Using a series of linear regression models applied to survey data collected in a diverse sample, we analyzed variation in both precision medicine knowledge and trust in biomedical research with socioeconomic factors as a way to understand the range of precision medicine knowledge (PMK) in a broadly representative group and its relationship to trust in research and demographic characteristics. Our results demonstrate that identifying as Black, while significantly PMK, explains only 1.5% of the PMK variance in unadjusted models and 7% of overall variance in models adjusted for meaningful covariates such as age, marital status, employment, and education. We also found a positive association between PMK and trust in biomedical research. These results indicate that race is a factor affecting PMK, even after accounting for differences in sociodemographic variables. Additional work is needed, however, to identify other factors contributing to variation in PMK as we work to increase diversity and inclusion in precision medicine applications.

## Introduction

Precision medicine (PM) is changing the one-size-fits-all healthcare paradigm for prevention and treatment of diseases through incorporation of an individual’s genetic makeup, environment, and lifestyle [1, 2]. At the same time, however, PM advances have the potential to widen racial and ethnic health disparities in the United States [3–7]. People of non-European ancestry are underrepresented in genetic databases, a sampling bias that can translate to clinical care bias and result in disparate outcomes or health disparities [8, 9]. Little progress has been made since Need and Goldstein reviewed GWAS studies in 2009 and found that participants of European ancestry outnumbered other races 10:1 [10]. As GWAS studies have expanded, we have also seen inequity in uptake of PM applications and services across racial groups [11–13]. Understanding why people are unaware of or choose not to participate in PM initiatives is critical to the success of PM. In general, many studies highlight negative attitudes of minorities towards PM approaches [14–16]. Yet some studies suggest there is an opportunity to influence attitudes positively to increase participation. Research summarized here suggests that factors other than race itself could explain these observed differences and therefore serve as potentially modifiable influencers of PM knowledge. Several explanations for these disparities are summarized here and include (1) attitudes toward PM and PM-related research topics like genetics and biobanks, (2) lack of awareness of PM research and care options, (3) trust in medical research, and (4) socioeconomic status. Like attitudes and awareness, precision medicine knowledge (PMK) is modifiable and could be contributing to the variation in PM uptake and the resulting disparities. Precision medicine knowledge (PMK) is health literacy in that it refers to factors contributing to a patient’s capacity to make informed decisions about their health care in a precision medicine context. This specific health literacy includes knowledge of basic terms plus the processing and understanding of certain concepts underlying precision medicine, such as how genes relate to health and health care and what ethical questions arise from genetic testing. An accurate understanding of these driving factors could reduce variation in uptake and the resulting disparities.

Genomic health literacy, though less studied to date, plays a role in PM engagement: from participating in research, to processing risk assessments and acting on PM-based medical advice, to participating in policy discussions about data use and the role of PM in health care [17]. Furthermore, differences in knowledge and attitudes toward PM were influenced by health literacy more than race and ethnicity [18, 19].

Medical mistrust is a significant influencer of attitudes towards genetic testing [20]. In a systematic review of barriers to minority research participation, 77% of articles reviewed cited medical mistrust as a barrier which held true across all four racial minorities studied (African Americans, Pacific Islander, Latino, Asian American) [21]. These differences are due to concerns that benefits will not be equitably distributed [22, 23], fear of being a “guinea pig,” [24, 25] and lack of legal protection for research subjects [26]. One study demonstrated that, despite similar attitudes towards the benefits of PM, Blacks were significantly more likely than Whites to be concerned about discrimination based on genetic results, use of genes and genetic information without consent, and costs to receive PM [27].

Recognized social determinants of health, such as income, Internet access, and numeracy skills also determined differences in PM awareness [18, 19]. Preferred source of medical information may also play a key role. Previous literature suggests that Hispanics are more likely to utilize the radio for medical information, while Blacks more frequently pay attention to television [28]. Other authors have suggested that improving health information delivery to minority populations has the potential to decrease disparities in care [29]. It is possible that different racial groups have varying exposure to informative and accurate PM concepts based on their preferred sources for health information.

Using data from the Mid-South Clinical Data Research Network (MS-CDRN) Consumer Interest and Attitudes Survey, we determined the contribution of socioeconomic factors to differences in overall PM knowledge and trust in biomedical research. Other data from this survey, published elsewhere, has compared the Hall and Mainous trust scales [30] to assess the relationship between race, health literacy, and values important when deciding about genetic testing [18]. In this study, we hypothesized that, after controlling for age, marital status, employment, and education, race itself would not be a predictor of PM knowledge, as the social construct of race masks underlying factors contributing to disparities in PM knowledge which, unlike race, could be addressed. The findings from this work may inform efforts to recruit minority participants into PM knowledge initiatives, therefore, benefiting individual and public health.

## Materials and methods

### Study population

This study was a cross-sectional survey of 3847 adult participants of the Mid-South Clinical Data Research Network (MS-CDRN), one of 11 CDRNs funded by the Patient-Centered Outcomes Research Institute [31]. The CDRN was established to facilitate involvement of patients in generating research questions and participating in research studies in order to further patient-centered research. In the MS-CDRN, clinical data from over 20 million patients are included from three large health systems encompassing 32 hospitals and hundreds of ambulatory practices. The health settings include academic medical centers, community-based hospitals, traditional outpatient clinics and federally-qualified health centers. Previous studies have included surveys of parent willingness to participate in HPV vaccination clinical trials and the relationship between depression and perceived health competence [31]. Our patient survey participants were recruited from June 2014 to June 2015 from this larger cohort. All study participants provided written informed consent prior to survey completion.

The research team identified priority populations consisting of racial/ethnic minorities, individuals with multiple chronic conditions, low-income groups, rural and urban residents, and older adults. Anyone over the age of 18 with the capacity to consent was invited to participate. Recruitment strategies included in-person engagement at community health centers, minority-owned barbershops, and community health fairs. In addition, online recruitment was utilized specifically through the Vanderbilt patient portal and ResearchMatch, a volunteer registry. Participants who completed the survey received a $10 compensation. This study was approved by the Vanderbilt Medical Center Institutional Review Board.

### Survey

Patients were asked to respond to a series of demographic questions, including educational level, household income, and race. To create racial groups of sufficient size for statistical comparison, participants were asked to select one of nine racial categories. The racial groups included in this analysis were Asian, Black, Hispanic, and White.

The survey was developed based on literature evidence of common concerns about PM. The survey instrument asked participants to rate their familiarity with PM terms first and then rank the importance of certain factors in guiding future research and healthcare on a five-point Likert scale (1 = not at all important; 5 = extremely important). As no validated tools existed to measure these concepts at the time of this study, we developed new instruments based on previous research conducted by the study team and a literature review of genetics literacy [32]. Experts were asked to assess the relevance and clarity of each item to measure its content and face validity. The third part of the survey was one of two different validated scales to measure trust in biomedical research. Participants completed all 12 questions on the respective assigned validated scale. Half of the participants selected at random received the Hall scale [33], while the other half received the Mainous scale [34]. This strategy was chosen to assess the consistency between the two surveys, and the results of this work are published elsewhere [30].

In addition, basic demographic information was also collected, including ethnicity, educational attainment, and household income. Barriers to participation in research were collected using a modified, 14-item scale developed by Mouton et al. [35]. The final item for measuring barriers to participation (“In my opinion, research in the United States is…”) had three possible response options: “ethical,” “not ethical,” and “I don’t know.” To preserve how this item loaded onto the overall score of the scale, the variable was scored such that “ethical” was recoded to 2.54, “not ethical” was recoded as 0.83, and “I don’t know” was recoded as 1.69. The total score across these 13 domains was calculated as a simple sum, with some items reversed scored so that higher scores represented a lower perception of participation barrier. The survey items are summarized in Table 1. The complete survey designed for use in this study is provided in the additional supporting information [Online Resources 1–4].

**Table 1:** Variables: Survey questions, items, and response modes.

Variable	Questions Asked	Response
Demographics	Age	Year of birth
	Race/Ethnicity	7 Choices + Other
	Sex	Male/Female/Other
	Marital Status	5 Choices
	Employment Status	6 Choices + Other
	Household Size	Free-text
	Number of comorbidities	6 Choices + Other and None
	Household Income	7 Choices
	Health Insurance	6 Choices + Other
Precision Medicine Vocabulary	How familiar are you with the following words or phrases?	5-point Likert Scale
	• Genetic testing	
	• Biological indicators/biomarkers	
	• Precision medicine	
	• Pharmacogenetics	
Precision Medicine Attitudes	My healthcare is specific to me. No two cases are the same.	5-point Likert Scale
	My genes can be used to determine the best treatment for me.	5-point Likert Scale
	My genes and other health information can be used to help prevent or treat health conditions in my family.	5-point Likert Scale
	My health information is kept private and secure.	5-point Likert Scale
	I have access to my own health records and can decide which health care providers and researchers have access to them.	5-point Likert Scale
	I can add information about my health to my records.	5-point Likert Scale
Sources of Medical Information	How much do you trust information about health or medical topics from each of the following?	9 Choices, each rated on a 5-point Likert Scale

### Variables

Precision medicine knowledge (PMK) is an continous outcome variable created as a sum score of 10 questions. Four questions asked participants about their familiarity with these PM terms: genetic testing, biological indicators/biomarkers, precision medicine, and pharmacogenetics. Participants were asked to state their familiarity with the terms on a Likert Scale (1 = not at all familiar, 5 = extremely familiar). An additional six questions asked participants about attitudes towards PM concepts. Examples of questions included: “My healthcare is specific to me. No two cases are the same.”; “My genes can be used to determine the best treatment for me.”; “My genes and other health information can be used to help prevent or treat health conditions in my family,”; “My health information is kept private and secure,”; “I have access to my own health records and can decide which health care providers and researchers have access to them.”; and “I can add information about my health to my health records.” Participants were asked to rate importance on a Likert scale (1 = not at all important, 5 = extremely important). A higher total PMK score indicated greater familiarity with terms and benefits, with a maximum score of 50.

To create an overall trust in biomedical research (TBR) variable, each participant’s score from the Hall survey [33] was standardized. Items 2, 4, 7, and 10 were reverse-coded prior to summation. This was repeated for the Mainous [34] participants, with items 1–6 and 12 reverse coded prior to summation. Higher scores demonstrate higher trust towards biomedical research, with a maximum score of 5. TBR scores were treated as continuous outcome variables in the models. Based on previous research using this database comparing results between the Hall and Mainous scales [30], we were able to compare overall TBR scores for participants, regardless of which survey they took. We standardized each score distribution by subtracting its mean and dividing by its standard deviation.

### Statistical analysis

Descriptive statistics were used to calculate demographic characteristics. For each outcome (PMK and TBR), we created a linear regression model to compare the racial group of interest and the contribution of race to the outcome. Predictive mean matching was used to account for missing data. Covariates (marital status, employment status, education, age, and gender) were selected *a posteriori*, based upon a review of relevant literature. Due to the association of race with health literacy and education, these were assessed as additional interaction variables in the model, with education coded as a continuous variable. Analysis was conducted in R (version 3.4.3).

## Results

Of the 3847 participants, 83% identified as White and 15% identified as Black. The mean age was 48 (standard deviation: ±16) years, 69% percent of the population identified as female, and 61% of participants had a college degree. Table 2 contains additional demographic information. Participants could state more than one race, allowing the total to be greater than 100%.

**Table 2:** Patient demographics.

Characteristic		n (%)
Total		3847 (100)
Race		
	Asian	84 (2.2)
	Black, African American, African, or Afro-Caribbean	594 (14.4)
	Hispanic, Latino, or Spanish origin	120 (3.1)
	Middle Eastern/North African	16 (0.4)
	Native American, American Indian, or Alaskan Native	71 (1.8)
	Native Hawaiian, Guamian, or Chamorro	8 (0.2)
	White	3192 (83.0)
	More than one race	124 (3.2)
	Other	22 (0.6)
	Missing	29 (0.8)
Sex		
	Male	1203 (30.2)
	Female	2744 (69)
	Other	3 (0.1)
	Prefer not to answer	7 (0.2)
	Missing	20 (0.5)
Marital Status		
	Now married	2196 (55.2)
	Separated	62 (1.6)
	Divorced	501 (12.6)
	Widowed	137 (3.7)
	Never married	771 (19.4)
	Living with a partner or significant other	258 (6.5)
	Prefer not to answer	33 (0.8)
	Missing	19 (0.5)
Highest degree or level of school		
	8th grade or less	26 (0.7)
	Some high school, but did not graduate	91 (2.3)
	High school graduate or GED	402 (10.1)
	Some college or 2-year degree	1042 (26.2)
	College graduate	1135 (28.5)
	More than a college degree	1242 (31.2)
	Prefer not to answer	21 (0.5)
	Missing	18 (0.5)
Employment Status		
	Employed Full Time	2012 (50.6)
	Employed Part Time	352 (8.9)
	Unemployed	217 (5.5)
	Volunteer	34 (0.9)
	Stay-at-home parent	177 (4.5)
	Retired	665 (16.7)
	Receiving Disability	279 (7)
	Other	223 (5.6)
	Missing	18 (0.5)

### Precision Medicine Knowledge (PMK)

The average PMK score was 38.20 out of a total of 50 for the 1142 participants who fully answered the questions. Average PMK by race was 36.16 (Black), 38.36 (White), 38.87 (Asian), and 38.31 (Hispanic). Self-identification as Black was negatively correlated with PMK when compared to all other participants. This relationship describes about 1.5% of the total variation in PMK (p < 0.001). Self-identification as Asian or Hispanic was not strongly correlated with PMK. The remaining three racial categories (Middle Eastern, Native American, and Native Hawaiian) could not be compared due to small sample size. When adjusting for covariates such as age, marital status, employment, and education, self-identification as Black was still negatively correlated with PMK, with the model explaining 6.7% of overall variance (p = 0.0026). There was no evidence that race modified the effect of education on PMK when including race and education as interaction terms in the model (p = 0.2681). The results of the regression analysis are summarized in Table 3.

**Table 3:** Precision medicine knowledge regression model results.

	ADJUSTED					UNADJUSTED				
	Estimate	Std. Error	t value	Pr(>|t|)		Estimate	Std. Error	t value	Pr(>|t|)	
	3.7638	0.1002	37.5632	0.0000	*					
										
Asian	0.0272	0.1586	0.1717	0.8637		0.8400	1.5500	0.5400	0.6000	
Black	-0.1765	0.0584	-3.0222	0.0026	*	-2.3100	0.5600	-4.4149	0.0000	*
Hispanic	0.0429	0.1484	0.2891	0.7726		0.4300	1.5500	0.2800	0.8000	
	0.1002	0.0181	5.5360	0.0000	*					
	-0.0001	0.0017	-0.0654	0.9479						
	0.0007	0.0414	0.0178	0.9858						
Separated	0.2597	0.1703	1.5251	0.1275						
Divorced	0.0551	0.0531	1.0387	0.2992						
Widowed	0.0796	0.1133	0.7028	0.4823						
Never Married	-0.1001	0.0559	-1.7904	0.0737						
Living with Partner/Significant Other	-0.0077	0.0880	-0.0873	0.9304						
Employed Part Time	0.1202	0.0717	1.6779	0.0937						
Unemployed	-0.0454	0.0969	-0.4681	0.6398						
Volunteer	0.0273	0.2227	0.1226	0.9024						
Stay-at-home Parent	-0.0032	0.0993	-0.0319	0.9746						
Retired	0.0123	0.0593	0.2074	0.8357						
Receiving Disability	-0.0243	0.0681	-0.3567	0.7214						
Other	0.0202	0.0830	0.2430	0.8080						

### Trust in Biomedical Research (TBR)

Self-identification as Black was negatively correlated with TBR when compared to White participants (p < 0.001). This relationship describes about 5.5% of the total variation in TBR. Self-identification as Hispanic or Asian was also negatively correlated with TBR, each explaining 0.1% of the variation. When adjusting for covariates such as age, marital status, employment, and education, self-identification as White was still positively correlated with TBR, and explained 6.8% of the overall variance. The results of the regression analysis are summarized in Table 4.

**Table 4:** Trust regression models.

		ADJUSTED					UNADJUSTED				
		Estimate	Std. Error	t value	Pr(>|t|)		Estimate	Std. Error	t value	Pr(>|t|)	
**Coefficient**		0.0561	0.0880	0.6369	0.5242						
**Race**							(0.3000)	0.1300	2.2000	0.0200	*
	Asian	(0.3146)	0.1372	(2.2930)	0.0219		(0.6600)	0.0440	14.9000	-	*
	Black	(0.6074)	0.0494	(12.2893)	-	*	(0.2800)	0.1300	2.2000	0.0270	*
	Hispanic	(0.4458)	0.1185	(3.7610)	0.0002	*					
**Education**		0.0204	0.0159	1.2859	0.1986						
**Age**		0.0009	0.0015	0.6182	0.5365						
**Female**		0.0288	0.0377	0.7628	0.4456						
**Marital Status**											
	Separated	0.0811	0.1330	0.6100	0.5419						
	Divorced	0.0820	0.0532	1.5416	0.1233						
	Widowed	0.0511	0.0936	0.5459	0.5851						
	Never Married	(0.1104)	0.0504	(2.1897)	0.0286	*					
	Living with Partner/Significant Other	0.1345	0.0702	1.9156	0.0555						
**Employment Status**											
	Employed Part Time	0.0297	0.0603	0.4919	0.6228						
	Unemployed	(0.1016)	0.0774	(1.3125)	0.1895						
	Volunteer	(0.2475)	0.1839	(1.3457)	0.1785						
	Stay-at-home Parent	(0.0362)	0.0848	(0.4267)	0.6697						
	Retired	(0.1469)	0.0570	(2.5800)	0.0099	*					
	Receiving Disability	0.0488	0.0691	0.7061	0.4802						
	Other	(0.0300)	0.0751	(0.3998)	0.6894						

Given that the analyzed covariates did not explain much of the variance in trust or PMK, we theorized that there may be a relationship between the two outcome variables and found a positive association between PMK and TBR (β = 0.1516, SE = 0.0512, p = 0.003).

### Preferred health information source

As our initially analyzed covariates did not explain the variance in trust or PMK, we explored the theory that information source preferences could differ widely within our population and could contribute to disparities in PMK that were not addressed by the previous models. The frequency results are displayed in Table 5. When asked about preferences for health information sources, the most popular answer was internet, with approximately 32% of respondents selecting this choice. Other popular sources included doctors (31%), family (15%), or friends (10%). Among racial subgroups, the rank order for information source preference remained the same. Of the participants who answered the question and identified as White, the most frequently preferred source of information was family (30%), followed by the internet (27%), and then doctors (26%).

**Table 5:** Information source by race.

	Family	Friend	Doctor	Internet	Radio, Newspaper, Magazines	Telephone	Alternative Provider	Other	Total*
**Asian**	29%	10%	22%	27%	5%	2%	3%	2%	292
**Black, African American, African, or Afro-Caribbbean**	34%	9%	23%	23%	6%	2%	2%	2%	1765
**Hispanic, Latino, or Spanish origin**	32%	7%	23%	26%	4%	2%	3%	3%	379
**Middle Eastern/North African**	25%	14%	22%	22%	5%	3%	5%	6%	65
**Native American, American Indian, or Alaskan Native**	28%	7%	24%	26%	4%	3%	5%	3%	254
**Native Hawaiian, Guamian, or Chamorro**	32%	8%	28%	24%	0%	4%	4%	0%	25
**White**	30%	8%	26%	27%	4%	1%	3%	1%	10754
**Other**	40%	4%	20%	29%	2%	0%	2%	4%	55

*Respondents can select more than one information source

## Discussion

Identifying strategies to address disparities in PM research and applications is imperative to the successful adoption of PM strategies. Results from the MS-CDRN Participant Survey, designed to collect data on stakeholder opinions about participation in that research network, have allowed us to identify gaps between race, socioeconomic barriers, and perceived mistrust on overall PMK. Black participants have significantly lower PMK and TBR, even after controlling for meaningful covariates. This is also true of Asian and Hispanic participants for TBR models. However, the models explain less than 7% in the overall variation in PMK, even after adjusting for meaningful covariates included in this survey, indicating that the survey data are an incomplete measure of factors related to PMK and TBR. Moreover, there is a significant association between PMK and TBR. The magnitude of these differences appears to be greater for Black participants compared to Hispanic or Asian participants. Additionally, the most popular health information sources (family, internet, and doctors) were consistent, regardless of race, and therefore are unlikely to contribute to the differences observed.

These results refuted our original hypothesis that the effects of race would be null after accounting for sociodemographic variables of education, income, employment, and marital status and confirms previous research citing race as a barrier for participation in biomedical research. The results from the models created in this study suggest that we still have an incomplete picture of factors influencing PMK. Until we understand why participants choose to not participate in PM initiatives or have not yet accepted the potential benefits, disparities in PM acceptance will grow. These factors could include previous interactions with the healthcare system [34], self-efficacy [36], and health literacy, specifically genetics literacy [37]. While our survey captures many social determinants of health, it is possible that additional drivers such as food insecurity, housing instability, or neighborhood safety issues may limit the benefits of PM in certain populations.

PM participation is modifiable, and early adopters have consistent personality traits, regardless of minority status [38]. Meaningful interventions, such as better representation of socioeconomic diversity in research leadership, tailored health education materials of appropriate literacy, and improved genetics education of the public, are all potential methods to decrease disparities in PM knowledge and attitudes which, in turn, could decrease differences in participation [39]. Furthermore, interventions that address logistical barriers, such as the complexity of payer coverage for genetic testing [40], the value of negative or unknown results to patients [41, 42], or the burden on primary care providers [43], is sorely needed to increase potential benefits of PM.

Previous research also supports these findings. A qualitative study found that African American focus group participants recommended a reduction in technical detail for genetics communication aids [44]. Both providers [45] and patients [46] recognize the need for increased genetics education to alleviate disparities in knowledge and attitudes towards PM. Culturally-tailored material and engagement of local stakeholders has been successful in improving recruitment of underrepresented groups in research [47]. Among Asian Americans, opportunities for improving participation include providing linguistically appropriate materials on genetic testing [48]. In addition, more granular details including specific countries of origin and length of time in the US could help researchers better understand differences within the Asian population as a whole [49].

In response to these and other studies, there is significant ongoing work to specifically address disparities in PM. One such example is the All of Us Research Program, which aims to increase genetic biodiversity by creating a national database [50]. This organization offers materials in a variety of different languages, has partnered with trustworthy community organizations to engage and retain participants, and utilized a patient advisory board early to advise development [51]. Meanwhile, The Personalized Medicine Research Project consulted patient participants in the design of the protocol to include changes such as newsletters to disseminate information to study participants, external advisory boards, and focus groups [52]. We believe that engaging participants in these novel ways can mitigate differences in PMK and TBR though the results of these initiatives are still being studied.

A strength of the current study is that the demographic distribution is similar to the population as a whole. In the analysis we found 15% of our 3847 participants identified as Black and in the entire Mid-South CDRN for 20,873,295 people, as of 2017, 17.4% identified as black or African American. Therefore, the percentage of black participants in our study properly reflects the percentage in the overall population [53]. Moreover, according to the 2010 US Census, which has its limitations, 13% of the US population identifies as Black, similar to the percentage of participants in this study [54]. Special efforts were made to recruit underrepresented groups to participate in this MS-CDRN Survey through recruitment at federally-qualified health centers and community settings (i.e. barber shops). The population’s median age was reported to be 56.3. 84.1% were White, 9.9% were Black, 1.8% were Hispanic, and Asian was not reported in the results. A predominance of females was also seen in the study population as a whole (64%) [55]. The surveys were comprehensive, including information on several variables impacting participation in PM, such as attitude, awareness, and trust in medical research, as well as several other confounding variables including age, marital status, employment, and education, which were successfully incorporated into statistical models. In addition, our study creates a systematic measure of PM knowledge consisting of a calculated value for PM-related health literacy plus the six foundational PM values. This metric can be replicated in future studies to quantitatively deepen our understanding of the factors driving PM use.

### Limitations

There are several limitations to this study. First, it relied on self-reported data and not objective measurements of PM knowledge. As no validated tools exist, the authors relied on methods that have been utilized in other genetics literature. Second, the sample was limited to those who electively participated in a survey about research attitudes. This resulted in a participant cohort with above average levels of education and income that was majority female. In addition, this nonresponse bias may have led to higher participation rates among people with higher TBR scores compared to the general population. For these reasons, these conclusions may not be generalizable to the whole population. Unfortunately, this study was not powered to address the nuances within race and can only draw conclusions generally about those who identify broadly as White, Black Asian, and Hispanic. These categories do not fully represent the spectrum of racial diversity.

## Conclusions

As the field of PM grows, we must be cautious about growing disparities in its understanding and dissemination. Without significant improvement in diversity and inclusion in genomic research, any advancements from PM applications addressing the disease burden can therefore be expected to accrue inequitably, favoring those of European ancestry over those of non-European ancestry [39]. While statistical models are able to capture individual factors such as education, income, and race it is clear that PM knowledge represents the end result of a complex, longitudinal interaction between providers, patients, the scientific community, society, and the environment. Future statistical models must incorporate additional factors to better understand variations in PM knowledge/awareness. Additional explorations could include topics such as use of genetic testing by law enforcement, access to direct-to-consumer testing, insurance access, and measures specific to science education, using validated tools if available. Efforts must be made to address sociocultural barriers as well as logistical barriers to accessing genomic testing. Equitable distribution of PM interventions and their benefits requires deepening our genomic knowledge in addition to our sociocultural knowledge.

## Supporting information

S1 File (PDF)

S2 File (PDF)

S3 File (PDF)

S4 File (PDF)
