# Supplementary material for: Factors influencing precision medicine knowledge and attitudes
Source: PLoS One. 2020 Nov 11;15(11):e0234833. doi: 10.1371/journal.pone.0234833 (PMC7657499; doi:10.1371/journal.pone.0234833)
Supplement: S1 File — (PDF) [file pone.0234833.s001.pdf]

Barriers To Participation In Research

The next set of questions asks you about potential barriers to participating in research. Again, by clicking submit, you acknowlege that your participation in this survey is voluntary and confidential.

Please indicate to what degree you agree or disagree with the following statements regarding participation in research.

	Strongly disagree	Disagree	Neither agree nor disagree	Agree	Strongly agree
Participation in clinical research benefits society	○	○	○	○	○
Participation will mean better care	○	○	○	○	○
Participation in research is risky	○	○	○	○	○
Researchers do not care about me	○	○	○	○	○
Participation in research is enjoyable	○	○	○	○	○
Participation in research allows me to socialize	○	○	○	○	○
Participation in research is against my religion	○	○	○	○	○
Participation in research is morally wrong	○	○	○	○	○
Transportation is a problem for people who participate	○	○	○	○	○
Scientists cannot be trusted	○	○	○	○	○
It is better to be treated by doctors who are researchers	○	○	○	○	○

Which of the following are barriers for you when considering participating in research? Check all that apply.	☐ Technical language on study materials (i.e. flyers, brochures, text messages, consent form, etc.)<br>☐ Cultural appropriateness of study materials (i.e. flyers, brochures, text messages, consent form, etc.)<br>☐ Burden to yourself or your family (i.e. time, cost, social acceptance)<br>☐ The feeling of being undervalued or poorly treated by health care system<br>☐ Limited access to research studies<br>☐ Personal attitude towards research in general<br>☐ Limited resources to participate in research (i.e. transportation, phone, internet, etc.)<br>☐ Lack of sensitivity from the researcher<br>☐ Lack of trust/mistrust/distrust<br>☐ Lack of motivation/willingness<br>☐ Fear<br>☐ Religious/spiritual beliefs<br>☐ Lack of awareness of studies (i.e. poor advertisement in your environment)<br>☐ The feeling that your health condition is stigmatized by society/health system/health providers (i.e. obesity, mental health, etc.)<br>☐ Restricted eligibility criteria (i.e. being turned away because you did not fit the screening criteria)<br>☐ Other
If other, please list:	________________________
In my opinions, research in the United States is	◯ Ethical<br>◯ Not ethical<br>◯ Don't know<br>◯ Other
If other, please describe.	______________________________

Thank you. Please hit submit below to continue to the final section of the survey.
