# Supplementary material for: Factors influencing precision medicine knowledge and attitudes
Source: PLoS One. 2020 Nov 11;15(11):e0234833. doi: 10.1371/journal.pone.0234833 (PMC7657499; doi:10.1371/journal.pone.0234833)
Supplement: S2 File — (PDF) [file pone.0234833.s002.pdf]

# Patient/Consumer Experience and Interest in Research

You have been selected to complete this research survey regarding your preferences, perspectives and willingness to participate in research. We are conducting this survey from the Vanderbilt University Medical Center to help us understand patient and consumer perspectives on participating in research.

The benefit of completing this survey is to inform researchs of patient and consumer perspectives on research and help identify approaches to research that are important to patients and consumers.

Your participation is strictly voluntary, and your individual responses will be kept anonymous. There is minimal risk, and refusing to participate will not impact your health care or opportunity to participate in future research. The survey will take about 15 minutes to complete.

All participants who complete the survey will receive $10 for their time and participation. Thank you!

| | |
|---|---|
| Please read the above statement. | ☐ I have read and understand the above statement, and I am aware that my participation is voluntary and anonymous. |
| E-mail. Please include your email address so that you may be contacted to receive your compensation. Your responses will not be identified to your email address. | ________________________________ |

## The first set of questions tells us about you and your background.

What is your year of birth?

______________________________

What is your race or ethnicity? (Check all that apply).

- ☐ Asian (i.e. Asian Indian, Chinese, Filipino, Japanese, Korean, Vietnamese, Hmong, Laotian, Thai, Pakistani, Cambodian, etc.)
- ☐ Black, African American, African, or Afro-Caribbean (i.e. African American, Haitian, Nigerian, etc.)
- ☐ Hispanic, Latino, or Spanish origin (i.e. Mexican, Mexican American, Puerto Rican, Cuban, Argentinian, Colombian, Dominican, Nicaraguan, Salvadorian, Spaniard, etc.)
- ☐ Middle Eastern/North African
- ☐ Native American, American Indian, or Alaskan Native (i.e. Navajo, Mayan, Tingt, etc.)
- ☐ Native Hawaiian, Guamanian or Chamorro, Samoan, Fijian, Tongan, etc.)
- ☐ White (i.e. German, Irish, Lebanese, Egyptian, etc.)
- ☐ Some other race or origin (please specify)
- ☐ Prefer not to answer

Other, please specify:

______________________________

What is your sex?

- ◯ Male
- ◯ Female
- ◯ Other
- ◯ Prefer not to answer

If other, please specify:

______________________________

What is your marital status?

- ◯ Now married
- ◯ Living with a partner or significant other
- ◯ Widowed
- ◯ Divorced
- ◯ Separated
- ◯ Never married
- ◯ Prefer not to answer

What is the highest degree or level of school you have completed?

- ◯ 8th grade or less
- ◯ Some high school, but did not graduate
- ◯ High school graduate or GED
- ◯ Some college or 2-year degree
- ◯ College graduate
- ◯ More than a college degree
- ◯ Prefer not to answer

Are you currently:

- ◯ Employed Full Time (32+ hours per week)
- ◯ Employed Part Time (less than 32 hours per week)
- ◯ Unemployed
- ◯ Volunteer
- ◯ Stay-at-home parent
- ◯ Retired
- ◯ Receiving disability
- ◯ Other

| Question | Response |
| --- | --- |
| If other, please describe: | ______________________ |
| How many people live in your home (including yourself)? | ______________________ |
| Have you visited your doctor in the past year? | ◯ Yes<br>◯ No |
| If yes, please select the clinic(s). | ☐ Vanderbilt University Medical Center<br>☐ Vanderbilt University Children's Hospital<br>☐ Vanderbilt University Stallworth Rehabilitation Hospital<br>☐ Vanderbilt University Psychiatric Hospital<br>☐ Vanderbilt University Outpatient Practices<br>☐ Meharry/Metro General Hospital<br>☐ Matthew Walker Community Health Center<br>☐ Prefer not to answer<br>☐ Other |
| If other, please list: | ______________________ |
| Do you have a cell phone? | ◯ Yes<br>◯ No |
| How often do you text? | ◯ Not at all<br>◯ Not often<br>◯ Somewhat often<br>◯ Often<br>◯ Very often |
| Can you access the internet using your phone? | ◯ Yes<br>◯ No |
| Do you have access to the internet at home? | ◯ Yes<br>◯ No |
| How confident are you using computers? | ◯ Very confident<br>◯ Confident<br>◯ Fairly confident<br>◯ Not confident<br>◯ Prefer not to say |

**Many patients have trouble understanding the medical  information they get at the hospital or doctor's office.**

How confident are you filling out medical forms by yourself?

- ◯ Extremely
- ◯ Quite a bit
- ◯ Somewhat
- ◯ A little bit
- ◯ Not at all

How often do you have someone help you read hospital materials?

- ◯ All of the time
- ◯ Most of the time
- ◯ Some of the time
- ◯ A little of the time
- ◯ None of the time

How often do you have problems learning about your medical conditions because of difficulty understanding written information?

- ◯ All of the time
- ◯ Most of the time
- ◯ Some of the time
- ◯ A little of the time
- ◯ None of the time

**The next few questions ask about your experience and feelings about working with numbers. For each of the following questions, please check the box that best reflects your answer.**

| | Not at all good/ Not at all often | Pretty good / Somewhat often | Good / Often | Very good / Very often | Extremely good/ Extremely often |
|---|---|---|---|---|---|
| How good are you at working with fractions? | ○ | ○ | ○ | ○ | ○ |
| How good are you at figuring out how much a shirt will cost if it is 25% off? | ○ | ○ | ○ | ○ | ○ |
| How often do you find numerical information to be useful? | ○ | ○ | ○ | ○ | ○ |

## How familiar are you with the following words or phrases?

| | Not at All Familiar | Slightly Familiar | Somewhat Familiar | Moderately Familiar | Extremely Familiar |
|---|---|---|---|---|---|
| Genetic Testing | ○ | ○ | ○ | ○ | ○ |
| Biological Indicators/Biomarkers | ○ | ○ | ○ | ○ | ○ |
| Precision Medicine | ○ | ○ | ○ | ○ | ○ |
| Pharmacogenetics | ○ | ○ | ○ | ○ | ○ |

**Some new approaches to preventing and treating health conditions will take into account people's individual variations in genes, environment, and lifestyle.**

**To help guide future research and healthcare, how important are the following to you?**

| | Not at all Important | Slightly Important | Somewhat Important | Moderately Important | Extremely Important |
|---|---|---|---|---|---|
| My healthcare is specific to me. No two cases are the same. | ◯ | ◯ | ◯ | ◯ | ◯ |
| My genes can be used to determine the best treatment for me | ◯ | ◯ | ◯ | ◯ | ◯ |
| My genes and other health information can be used to help prevent or treat health conditions in my family | ◯ | ◯ | ◯ | ◯ | ◯ |
| My health information is kept private and secure | ◯ | ◯ | ◯ | ◯ | ◯ |
| I have access to my own health records and can decide which health care providers and researchers have access to them | ◯ | ◯ | ◯ | ◯ | ◯ |
| I can add information about my health to my health records | ◯ | ◯ | ◯ | ◯ | ◯ |

Do you have or have you ever had any of the following long-term health conditions? (Please check all that apply).

- ☐ No health conditions
- ☐ High blood pressure
- ☐ Heart disease
- ☐ Heart attack
- ☐ Overweight or obese
- ☐ Diabetes
- ☐ Asthma
- ☐ Sickle Cell Disease
- ☐ Other
- ☐ Prefer not to say

If other, please specify:

__________________________________

Household Income (Optional)

- ◯ Less than $10,000
- ◯ $10,000 - $14,999
- ◯ $15,000 - $24,999
- ◯ $25,000 - $34,999
- ◯ $35,000 - $49,999
- ◯ $50,000 - $74,999
- ◯ $75,000 - $99,999
- ◯ $100,000 - $149,999
- ◯ $150,000 or more

| | |
|---|---|
| Health Insurance Category (Optional) | ◯ Insured<br>◯ Uninsured (No Insurance)<br>◯ Medicaid (TennCare)<br>◯ Medicare<br>◯ Self Pay<br>◯ Other |
| If other, please specifiy: | __________________________ |

**The next set of questions tells us about your experience with research (if any).**

Prior to today, have you ever been asked to participate in research?
- ◯ Yes
- ◯ No

Prior to today, have you ever participated in health-related research?
- ◯ Yes
- ◯ No

If yes, how would you describe the research? (select all that apply)
- ☐ A study comparing treatments or prevention methods (clinical trial)
- ☐ Testing to determine if you are high-risk for a certain disease
- ☐ Testing to determine if a condition has been passed down in your family (inherited)
- ☐ In-person focus group, facilitated discussion or survey of attitudes, beliefs or behaviors
- ☐ An online questionnaire or survey of attitudes, beliefs or behaviors
- ☐ Blood, tissue or other body fluid was collected for use in any current or future research
- ☐ Other

Other, please describe.

__________________________________

In general, what would be the preferred contact methods to learn about potential research studies? Select all that apply. (This is to help us gauge most appropriate methods; you will not be contacted based on your answers to this question).
- ☐ E-mail
- ☐ Cell phone text messaging
- ☐ Social media (such as Facebook or Twitter)
- ☐ Letter or postcard in the mail
- ☐ A computer-created phone message
- ☐ Personal phone call from research staff or my doctor
- ☐ Talking face-to-face with research staff or my doctor when I am visiting the clinic
- ☐ Other
- ☐ I am not interested in being contacted about future research studies

Other, please specify.

__________________________________

**If a research was studying a condition or health problem that you care about, would you participate if it required ...**

**(Please pick your level of interest by checking one of the options for each line below.**

| | Not interested | Somewhat interested | Very interested |
|---|---|---|---|
| Completing a survey two or more times | ○ | ○ | ○ |
| Giving a blood sample | ○ | ○ | ○ |
| Taking part in a study that involves talking by phone or over the internet (for example, to get advice about your health) | ○ | ○ | ○ |
| Taking part in a study in which you have to take a medication | ○ | ○ | ○ |
| Taking part in a study that involves meeting at a local community center or school | ○ | ○ | ○ |
| Taking part in a study that involves you and other people in your family | ○ | ○ | ○ |
| Taking part in a study in which you would stay in the hospital for one or more days | ○ | ○ | ○ |

What sources do you most often use to find information about health or medical topics? Check all that apply.

- ☐ Family
- ☐ Friend/Co-worker
- ☐ Doctor of other healthcare provider
- ☐ Internet
- ☐ Radio, newspaper or magazines
- ☐ Telephone information number of disease-focused group such as the American Cancer Society or the American Heart Association
- ☐ Complementary, alternative or unconventional practitioner
- ☐ Other
- ☐ I have never looked for information about health or medical topics

Other, please describe

__________________________________

**In general, how much do you TRUST information about health or medical topics from each of the following?**

| | Not at all | A little | Some | A lot |
|---|---|---|---|---|
| Doctor | ○ | ○ | ○ | ○ |
| Other healthcare provider (nurse, pharmacist or other professional who provides care) | ○ | ○ | ○ | ○ |
| Family or friends | ○ | ○ | ○ | ○ |
| Online community for patients or caregivers dealing with the same health condition | ○ | ○ | ○ | ○ |
| Disease-focused groups such as the American Cancer Society or the American Heart Association | ○ | ○ | ○ | ○ |
| Your health insurance company (health plan) | ○ | ○ | ○ | ○ |
| Internet | ○ | ○ | ○ | ○ |
| Television, radio, newspaper or magazines | ○ | ○ | ○ | ○ |
| Government health agencies | ○ | ○ | ○ | ○ |

Thank you. Please hit submit below to continue to the next part of the survey.
