# Supplementary material for: Factors influencing precision medicine knowledge and attitudes
Source: PLoS One. 2020 Nov 11;15(11):e0234833. doi: 10.1371/journal.pone.0234833 (PMC7657499; doi:10.1371/journal.pone.0234833)
Supplement: S3 File — (PDF) [file pone.0234833.s003.pdf]

Trust in Medical Research

The last set of questions will help us understand your level of trust in research and researchers in general. Again, by clicking submit, you acknowlege that your participation in this survey is voluntary and confidential.

The following twelve questions ask about your views on research. There are no right or wrong answers. For each statement below, please indicate how strongly you agree or disagree with it.

	Strongly disagree	Disagree	Neutral	Agree	Strongly agree
Doctors who do medical research care only about what is best for each patient	○	○	○	○	○
Medical researchers treat people like "guinea pigs"	○	○	○	○	○
It's safe to be in a medical research study	○	○	○	○	○
Some doctors do medical research for selfish reasons	○	○	○	○	○
Doctors tell their patients everything they need to know about being in a research study	○	○	○	○	○
A doctor would never ask me to be in a medical research study if the doctor thought there was any chance it might harm me	○	○	○	○	○
There are some things about medical research that I do not trust at all	○	○	○	○	○
A doctor would never recommend something that is not the best treatment just so he or she can study how it works	○	○	○	○	○
Medical researchers have no selfish reasons for doing research studies	○	○	○	○	○
Medical researchers do not tell people everything they really need to know about being in a research study	○	○	○	○	○
The only reason doctors do medical research is to help people	○	○	○	○	○

I completely trust doctors who do medical research	○	○	○	○	○

Thank you for your input. Please let us know if you would like to receive more potential surveys in the future.

Would you be willing to receive any follow-up surveys to your e-mail address in the future?

○ Yes
○ No
