# Supplementary material for: Factors influencing precision medicine knowledge and attitudes
Source: PLoS One. 2020 Nov 11;15(11):e0234833. doi: 10.1371/journal.pone.0234833 (PMC7657499; doi:10.1371/journal.pone.0234833)
Supplement: S4 File — (PDF) [file pone.0234833.s004.pdf]

Trust In Medical Research

The last set of questions will help us understand your level of trust in research and in researchers in general. Again, by clicking submit, you acknowlege that your participation in this survey is voluntary and confidential.

The following questions ask about your views on research. There are no right or wrong answers. For each statement below, please indicate how strongly you agree or disagree with it.

	Strongly disagree	Disagree	Neutral	Agree	Strongly agree
To get people to take part in a study, medical researchers usually do not explain all of the dangers about participation	○	○	○	○	○
Participants should be concerned about being deceived or misled by medical researchers	○	○	○	○	○
Usually, researchers who make mistakes try to cover them up	○	○	○	○	○
Medical researchers act differently toward minority subjects than toward white subjects	○	○	○	○	○
Medical researchers unfairly select minorities for their most dangerous research studies	○	○	○	○	○
Some medical research projects are secretly designed to expose minority groups to diseases such as AIDS	○	○	○	○	○
Medical researchers are generally honest in telling participants about different treatment options available for their conditions	○	○	○	○	○
Usually, medical researchers tell participants everything about possible dangers	○	○	○	○	○
All in all, medical researchers would not conduct experiments on people without their knowledge	○	○	○	○	○

Most medical researchers would not lie to people to try to convince them to participate in a research study	◯	◯	◯	◯	◯
In general, medical researchers care more about doing their research than about the participants' medical needs	◯	◯	◯	◯	◯
Researchers are more interested in helping their careers than in learning about health and disease	◯	◯	◯	◯	◯

Thank you for your input. Please let us know if you would like to receive more potential surveys in the future.

Would you be willing to receive any follow-up surveys to your e-mail address in the future?

◯ Yes
◯ No
